# Longitudinal Neuropsychiatric Symptom Trajectories in Frontotemporal Dementia: From Presymptomatic to Symptomatic Conversion

**DOI:** 10.1002/alz.090036

**Published:** 2025-01-03

**Authors:** Hyunwoo Lee, Imogene M Scott, Atri Chatterjee, Ian R MacKenzie, Maria I. Lapid, Edward D. Huey, Maria Carmela Tartaglia, Kejal Kantarci, Katherine P. Rankin, Howard J. Rosen, Brad F. Boeve, Adam L. Boxer, Ging‐Yuek Robin Hsiung, the ALLFTD Consortium

**Affiliations:** ^1^ University of British Columbia, Vancouver, BC Canada; ^2^ The University of British Columbia, Vancouver, BC Canada; ^3^ Mayo Clinic, Rochester, MN USA; ^4^ Brown University, Providence, RI USA; ^5^ Tanz Centre for Research in Neurodegenerative Diseases, University of Toronto, Toronto, ON Canada; ^6^ Department of Radiology, Mayo Clinic, Rochester, MN USA; ^7^ Memory and Aging Center, UCSF Weill Institute for Neurosciences, University of California, San Francisco, San Francisco, CA USA; ^8^ Memory and Aging Center, UCSF Weill Institute for Neurosciences, San Francisco, CA USA; ^9^ Department of Neurology, Mayo Clinic, Rochester, MN USA

## Abstract

**Background:**

Frontotemporal dementia (FTD) presents with heterogeneous neuropsychiatric symptoms (NPS). These symptoms often begin prior to the onset of FTD, and progress throughout the prodromal stages of FTD. Particularly, familial FTD due to autosomal dominant genetic mutations might display mutation‐specific NPS profiles. We hypothesized distinct NPS trajectories for chromosome 9 open reading frame 72 (*C9orf72*), progranulin (*GRN*), and microtubule‐associated protein tau (*MAPT*) mutation carriers during their transition from presymptomatic to symptomatic stages of FTD.

**Method:**

In this study, we analyzed N = 1662 participants from the ARTFL‐LEFFTDS Longitudinal FTLD (ALLFTD) Study, with 342 *C9orf72*, 148 *GRN*, 168 *MAPT* mutation carriers, and 1004 noncarriers. We used the CDR plus NACC FTLD global scores to define the conversion status, and stratified participants into four stages of progression: 1) Presymptomatic (CDR = 0 throughout the follow‐up), 2) Early conversion (began with CDR = 0, then increased to 0.5), 3) Advanced conversion (began with CDR = 0.5, then increased to 1.0 or above), and 4) Symptomatic (CDR>1.0 throughout). Over up to seven visits, the Neuropsychiatric Inventory Questionnaire (NPI‐Q) assessed changes in NPS. We analyzed total NPI‐Q score trajectories using a generalized linear mixed‐effects model, adjusting for age and baseline NPI‐Q scores.

**Result:**

Our findings showed similar NPS trajectories among carriers and noncarriers during presymptomatic stages. However, in the early conversion stage, *C9orf72* and *GRN* carriers exhibited significantly higher NPI‐Q score increases compared to *MAPT* carriers, primarily in the psychosis and hyperactivity domains. In the advanced and symptomatic stages, the rate of increase in NPS was not significantly different across groups.

**Conclusion:**

This study suggests that people with familial FTD, particularly those predicted to have underlying TDP‐43 pathology, may experience more severe neuropsychiatric symptoms like psychosis or hyperactivity as they progress from presymptomatic to prodromal phases. This trajectory appears distinct from those with tau pathology or sporadic FTD. Further studies are warranted to understand these unique progression patterns and their implications for FTD management.

